# Differences in the Subcortical White Matter Integrity Based on the Disorder of Consciousness at the Chronic Stage of Diffuse Axonal Injury: A Tract-Based Spatial Statistics Study

**DOI:** 10.3390/medicina62091708

**Published:** 2026-09-06

**Authors:** Sung Ho Jang, Min Jye Cho, Dong Hyun Byun

**Affiliations:** 1 Department of Physical Medicine and Rehabilitation, College of Medicine, Yeungnam University, 170 Hyeonchung-ro, Namku, Daegu 42415, Republic of Korea; strokerehab@hanmail.net (S.H.J.); ctfmon00617@daum.net (M.J.C.); 2 Department of Special Creative Convergence, College of Rehabilitation Sciences, Daegu University, 201 Daegu-Daero, Jillyang-eup, Gyeongsan-si 38453, Republic of Korea

**Keywords:** subcortical white matter, consciousness, diffuse axonal injury, tract-based spatial statistics, diffusion tensor imaging

## Abstract

**Highlights:**

**What are the main findings?**

- Tract-based spatial statistics (TBSS) revealed that DAI patients with a disorder of consciousness (DOC) exhibit significantly lower fractional anisotropy (FA) values in 44 of 48 subcortical white matter regions compared to non-DOC patients.
- Extensive microstructural degradation was confirmed globally, as the DOC group demonstrated significantly lower FA values across all 48 regions when compared to healthy controls.

**What are the implications of the main findings?**

- The severity of impaired consciousness in chronic DAI directly reflects the extent of microstructural injury across broad subcortical neural networks, rather than localized focal damage.
- Widespread subcortical white matter degradation disrupts the complex interplay of diverse neural networks (e.g., thalamocortical projections, ARAS, fronto-parietal networks) necessary for conscious perception, providing a crucial neuroanatomical basis for understanding consciousness disorders following DAI.

**Abstract:**

*Background and Objectives*: In this study, we investigate the relationship between the state of consciousness and the whole white matter integrity at the chronic stage in diffuse axonal injury (DAI) patients, using tract-based spatial statistics (TBSS). *Materials and Methods*: Twenty-seven chronic DAI patients and 22 healthy controls were enrolled. Patients were divided into a disorder of consciousness (DOC) group (*n* = 11, Coma Recovery Scale-Revised [CRS-R] score < 23) and a non-DOC group (*n* = 16, CRS-R score = 23). Fractional anisotropy (FA) values across 48 subcortical white matter regions of interest (ROIs), defined by the Johns Hopkins University atlas, were analyzed using TBSS to evaluate group differences. *Results*: The groups significantly differed on FA values of 48 ROIs (Pillai’s trace = 1.000, *p* < 0.05). The Tukey’s HSD test revealed that the FA values of 44 ROIs (except for the column and body of fornix, right corticospinal tract, left retrolenticular part of internal capsule, and left uncinate fasciculus) among the 48 ROIs of the DOC group were significantly lower than those of the non-DOC group, whereas the FA values of all 48ROIs of the DOC group were significantly lower than those of the control group (*p* < 0.05). On the other hand, except for the bilateral corticospinal tract, the FA values of 46 ROIs among the 48 ROIs of the non-DOC group were significantly lower than those of the control group (*p* < 0.05). *Conclusions*: In DAI patients, the state of consciousness was related to injury severity in most subcortical neural structures.

## 1. Introduction

Acquired physical and cognitive disabilities following traumatic brain injury (TBI) frequently manifest with severe consciousness disorders as a primary clinical complication [1]. The incidence of impaired consciousness, including coma, has been reported to range between two and ten per 100,000 patients following TBI [2,3,4]. Approximately 40~50% of all patients admitted to a hospital for TBI have been reported to suffer from diffuse axonal injury (DAI), and this is the most common cause of the vegetative state [5,6,7,8]. DAI represents widespread axonal damage caused by shearing forces from the acceleration, deceleration, or rotation of the brain during accidents, and it is typically diagnosed when the loss of consciousness lasts more than six hours after the trauma [9,10,11]. The pathology of DAI is characterized by widespread axonal injury involving multiple brain regions including the subcortical white matter, corpus callosum, upper brainstem, hippocampus, fronto-temporal lobe and their adjacent brain areas [4,7,12,13]. This explains why DAI is the commonest cause of progression to a vegetative state after TBI [6,7,8]. Therefore, elucidation of the neural structures related to impaired consciousness following DAI is clinically important for neuromodulation using repetitive transcranial magnetic stimulation (TMS) and transcranial direct current stimulation (TDCS), as well as for prognosis prediction [14,15].

Although the neural mechanisms of the human brain related to the control of consciousness have not been clearly elucidated, neural networks including the ascending reticular activating system (ARAS), the fronto-parietal network, the mesocircuit model, and the salience network have been reported to play a role in the control of consciousness [16,17,18,19,20,21]. Among the key known brain networks related to consciousness, the default mode network (DMN, connectivity between the medial prefrontal cortex and posterior cingulate cortex/precuneus) which is part of the fronto-parietal network plays a role in intrinsic awareness [22,23]. In addition, diverse subcortical structures such as the basal ganglia, thalamus, and brainstem communicate with broad cortical regions and contribute to arousal and consciousness [24]. In particular, the ARAS, which originates from the upper brainstem reticular nuclei to the thalamus (intralaminar nuclei) and cerebral cortex, is responsible for regulating arousal and wakefulness [25,26]. Many studies have reported a relationship between the level of consciousness and injury to local subcortical white matter structures, mainly in the brainstem, thalamus, and corpus callosum, in DAI patients [27,28,29,30,31,32,33]. In contrast, the whole subcortical structures related to impaired consciousness following DAI have not been clearly defined.

Diffusion tensor imaging (DTI), an advanced magnetic resonance imaging (MRI) technique, has the advantage of detecting whole-microstructural white matter abnormalities that are not generally identified in conventional brain MRI [34,35]. The characteristics seen through DTI provide information on white matter tract integrity by evaluating the diffusion of water molecules in its microstructure [36,37]. Among the various DTI analytic methods, tract-based spatial statistics (TBSS) is an automated, sensitive technique that can be used to conduct precise voxel-based white matter analysis of multi-subject diffusion imaging data [38]. Because it allows objective selection of regions of interest (ROIs), TBSS is a useful method that provides comprehensive information on global changes in subcortical white matter microstructure [38]. One TBSS-based study reported a correlation between the Glasgow Coma Recovery Scale and fractional anisotropy (FA) values of the posterior thalamic radiation and the superior cerebellar peduncle at the early stage of DAI [39]. However, there has been no study on the relation between consciousness and the subcortical white matter structure at the chronic stage of DAI.

In this study, we investigated the relationship between the state of consciousness and whole white matter integrity at the chronic stage in DAI patients, using TBSS.

## 2. Methods

### 2.1. Subjects

A total of 27 patients with DAI (24 men, 3 women; mean age 47.00 ± 15.89 years; range, 20~74), and 22 age-and gender-matched normal control subjects (11 men, 11 women; mean age 41.73 ± 12.36 years; range, 21~63) with no history of neurological/psychiatric disease were recruited for this study. The 27 patients with DAI were recruited according to the following inclusion criteria: (1) First ever TBI; (2) Impaired consciousness [Glasgow Coma Scale (GCS) score < 15 and Coma Recovery Scale-Revised (CRS-R) score < 23] at the onset of TBI; (3) Age 20–79 years; (4) Loss of consciousness (LOC) following the trauma lasting six hours or more; (5) conventional MRI showing no focal lesions other than DAI-specific microhemorrhages or gliosis; (6) DTI scans obtained at a chronic stage (more than four weeks after onset); and (7) no prior neurological or psychiatric history. The Yeungnam University Hospital Institutional Review Board approved this study (YUMC 2021-03-014-005). The demographic and clinical data of the patient and control groups are shown in Table 1. The patients with DAI who met the inclusion criteria were categorized according to the CRS-R score at the chronic stage: 11 (40.74%) of the 27 patients (8 men, 3 women; mean age 52.55 ± 16.24 years) whose chronic CRS-R score was below 23 were categorized in the disorder of consciousness (DOC) group, and 16 (59.26%) of the 27 patients (16 men; mean age 43.19 ± 14.96 years) whose chronic CRS-R score was 23 in the non-DOC group.

### 2.2. Evaluation of Consciousness Function

Consciousness was quantified using the CRS-R, a validated instrument encompassing six neurobehavioral subscales: arousal, auditory, communication, oromotor, motor, and visual [40,41]. The total score range is 0–23, and a lower score indicates a higher severity of consciousness disorder [40].

### 2.3. Diffusion Tensor Imaging

DTI was performed at a mean of 6.94 ± 6.36 months post-trauma using a 1.5-T Philips Gyroscan Intera scanner (Hoffman-LaRoche, Best, The Netherlands). A single-shot echo-planar imaging sequence was applied with 32 non-collinear diffusion-sensitizing gradients. Sixty-five contiguous slices were acquired parallel to the anterior–posterior commissure plane. Acquisition parameters wereas follows: acquisition matrix = 96 × 96, reconstructed to matrix = 192 × 192 matrix, field of view = 240 × 240 mm^2^, TR = 10,398 ms, TE = 72 ms, parallel imaging reduction factor (SENSE factor) = 2, echo planar imaging (EPI) factor = 59 and b = 1000 s/mm^2^, number of excitations (NEX) = 1, slice gap = 0, and a slice thickness of 2.5 mm.

### 2.4. Tract-Based Spatial Statistics

All data analyses were conducted utilizing the Functional Magnetic Resonance Imaging of the Brain (FMRIB) Software Library (FSL, version 6.0.5). Prior to TBSS analysis, diffusion-weighted images underwent standard preprocessing. Specifically, eddy current-induced image distortions and head motion were corrected using affine registration to the reference b = 0 volume. Non-brain tissues were then removed using the Brain Extraction Tool (BET). Subsequently, the diffusion tensor model was fitted at each voxel using FMRIB’s Diffusion Toolbox (FDT) to generate individual fractional anisotropy (FA) maps, based on a previously established method [38]. For the voxel-wise statistical evaluation of the FA data, we applied the Tract-Based Spatial Statistics (TBSS) tool implemented in FSL [42]. The FA data of all participants were registered into a common space through a nonlinear registration algorithm (www.doc.ic.ac.uk/~dr/software (accessed on 29 August 2021). We then generated a mean FA image and thinned it to construct a mean FA skeleton, which indicates the centers of all tracts common to the group members. Prior to entering the data into the voxel-wise statistics, the mean FA skeleton was binarized by applying a threshold of FA > 0.2. Each subject’s aligned FA data were subsequently projected onto this mean skeleton. Voxel-wise cross-subject statistics were then calculated to evaluate the differences in FA values among the groups. To correct for multiple comparisons, the family-wise error rate was controlled following threshold-free cluster enhancement. To further explore the data, we selected the voxels demonstrating significant differences in each tract based on the TBSS results, and computed the mean FA value for individual subjects. In order to summarize the FA data within a conventional neuroanatomical framework, the mean FA was calculated both across the entire skeleton and within 48 ROIs. These ROIs were defined by the intersections of the skeleton with the probabilistic Johns Hopkins University white matter atlases.

### 2.5. Statistical Analysis

Statistical analysis was performed using SPSS 21.0 for Windows (SPSS, Chicago, IL, USA). Multivariate analysis of variance (MANOVA) was performed to evaluate significant differences in the FA values of 48 ROIs between the DOC, non-DOC, and control groups, using Pillai’s trace, which yields a positive-valued statistic for model contribution; *p* < 0.05 was considered statistically significant. Additionally, to control for the confounding effect of age, a univariate analysis of covariance (ANCOVA) was performed on the whole-skeleton average FA value with age as a covariate, followed by Bonferroni post-hoc comparisons. To evaluate diagnostic relevance, a Receiver Operating Characteristic (ROC) curve analysis was conducted. Diagnostic power was assessed by calculating the Area Under the Curve (AUC), sensitivity, and specificity of the whole-skeleton average FA value for classifying patients into the DOC or non-DOC groups.

## 3. Results

**Figure 1:**
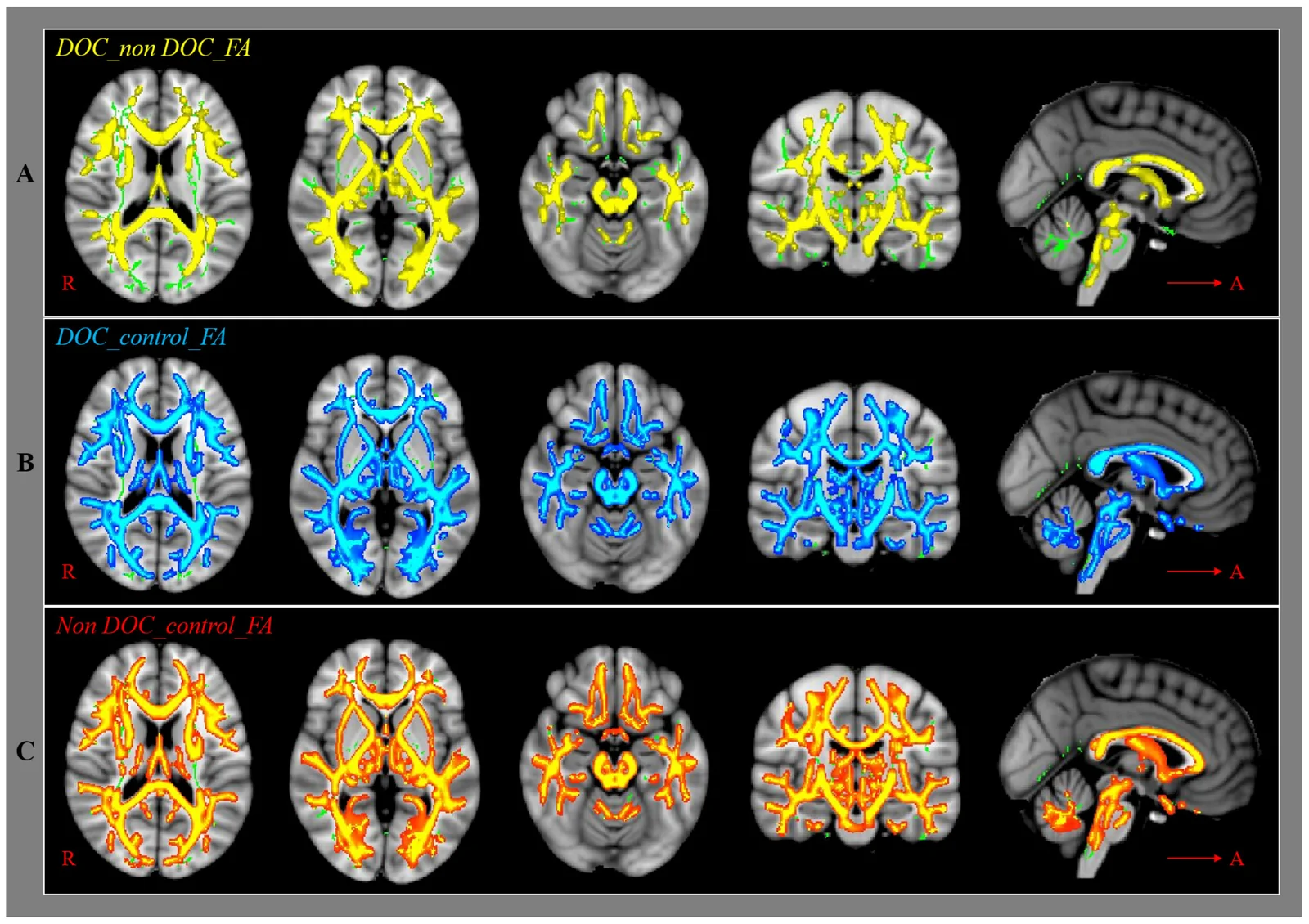
Results of tract-based spatial statistics analyses comparing the fractional anisotropy (FA) values of the 48 regions of interest (ROIs) in the disorder of consciousness (DOC), non-DOC, and control groups using a standard template of Johns Hopkins University diffusion tensor imaging-based white matter atlas. (**A**) The yellow voxels indicate areas with higher mean FA values in the non-DOC group than the DOC group except for the column and body of fornix, right corticospinal tract, left retrolenticular part of internal capsule, and left uncinate fasciculus. (**B**) The blue voxels represent the mean FA values of all 48 ROIs that are higher in the control group than in the DOC group. (**C**) The red voxels show areas with higher mean FA values in the control group than the non-DOC group except for the bilateral corticospinal tract.

The FA values for each group (DOC, non-DOC, and control) are summarized in Table 2. The groups significantly differed in the FA values of 48 ROIs (Pillai’s trace = 1.000, *p* < 0.05). The Tukey’s Honestly Significant Difference (HSD) test revealed that the FA values of 44 ROIs (except for the column and body of fornix, right corticospinal tract, left retrolenticular part of internal capsule, and left uncinate fasciculus) among the 48 ROIs of the DOC group were significantly lower than those of the non-DOC group. In contrast, the FA values for all 48 ROIs in the DOC group were significantly lower than those in the control group (*p* < 0.05). On the other hand, except for the bilateral corticospinal tract, the FA values of 46 out of the 48 ROIs of the non-DOC group were significantly lower than those of the control group (*p* < 0.05) (Figure 1). An additional univariate ANCOVA, adjusting for age, was performed on the whole-skeleton average FA value. This revealed a significant main effect of the group (F = 66.193, *p* < 0.001). Bonferroni post-hoc tests showed that the age-adjusted average FA of the DOC group (0.370 ± 0.007) was significantly lower than those of the non-DOC (0.413 ± 0.006) and control (0.467 ± 0.005) groups (all *p* < 0.001). This confirms that the global white matter degradation in the DOC group is independent of age. Finally, ROC curve analysis demonstrated that the whole-skeleton average FA value significantly differentiated the DOC and non-DOC groups (AUC = 0.875, 95% CI: 0.742–1.000, *p* = 0.001). At an optimal FA cutoff of 0.4050, the sensitivity and specificity for identifying the DOC group were 90.9% and 62.5%, respectively. Additionally, to explore the potential influence of sex on white matter integrity, we assessed sex differences in the whole-skeleton average FA exclusively within the control group. The analysis revealed no significant difference in the FA values between males and females (0.4627 ± 0.0190 vs. 0.4709 ± 0.0197; *p* > 0.05).

## 4. Discussion

This study investigated the relationship between the severity of impaired consciousness and whole subcortical white matter integrity at the chronic stage of DAI. The results are summarized as follows: (1) FA values of 44 out of the 48 ROIs assessed were lower in the DOC group than in the non-DOC group, (2) FA values of all 48 ROIs were lower in the DOC group than those in the control group, (3) FA values of 46 out of the 48 ROIs were lower in the non-DOC group than those in the control group.

The FA value is a common diffusion tensor tractography (DTT) parameter that reflects the integrity of white matter microstructure based on the degree of directionality of water diffusion [43,44]. Therefore, a decrease in the FA value of a neural structure indicates microstructural damage to white matter, including demyelination and axonal damage [45]. In this study, FA values were lower in 44 of the 48 ROIs in the DOC group compared with the non-DOC group. FA values of 46 out of the 48 ROIs were lower in the non-DOC group than those in the control group, suggesting more severe damage to the extensive subcortical white matter microstructure in the DOC group than in the non-DOC group at the chronic stage.

The thalamus plays a critical role in mediating consciousness, including attention and arousal. Specifically, the neural networks from the thalamocortical projections are reported to be important for sustaining normal consciousness [39,46,47,48]. The thalamocortical projections of the ARAS extend between the thalamic intralaminar nuclei and the frontal and parietal cortex regions via the internal capsule and corona radiata [49]. Specifically, these projections of the ARAS activate cortical neurons by contributing neuronal inputs to the anterior forebrain mesocircuit and the fronto-parietal network [50].

The fronto-parietal network consists of the DMN and the external network [50]. The DMN and the external network are responsible for the crucial roles of intrinsic awareness, and attention and action selection, respectively [22,23,50]. The medial prefrontal cortex and posterior cingulate cortex/precuneus, and the retrosplenial cortex and medial temporal lobe are known to structurally and functionally connect in a DMN [51,52]. With respect to the fronto-parietal network-related neural structures, the uncinate fasciculus and superior longitudinal fasciculus have been reported to support neuronal activity in the DMN and the external network [53].

Regarding the brainstem, the ARAS originates in the pontine reticular formation and connects with the central thalamus and cerebral cortex [50]. As the ARAS is known to activate the cerebral cortex and mediate consciousness functionally, the brainstem is reported to play a key role in alertness [54,55,56]. Given the brainstem’s association with consciousness, injury to brainstem-related fibers could impair consciousness [39,54,55]. In particular, the pontine crossing tract is a key nerve pathway in the lower dorsal ARAS which links the pontine reticular formation to the thalamic intralaminar nuclei [57].

Among the white matter structures most affected by DAI, the corpus callosum is the large white matter bundle that connects the two cerebral hemispheres [58]. The corpus callosum is a major white matter structure that is strictly associated with the integration and synchronization of interhemispheric sensorimotor and cognitive functions, and these processes are the foundation of conscious perception [59,60]. Consequently, these diverse subcortical structures have been reported to relate to consciousness. The widespread axonal injury seen in DAI, including the whole subcortical white matter, encompassing the thalamus, brainstem, cingulate gyrus, hippocampus, corpus callosum, and their adjacent brain areas, confirms the role of these structures in consciousness [12,13].

Regarding the generation of consciousness, the neural correlates of consciousness are the mechanisms that produce minimal consciousness for experiencing conscious perception [61,62]. The neural correlates of consciousness are distributed across the whole brain and interact through neural networks and loops, such as the thalamocortical projections, the fronto-parietal network, and the anterior forebrain mesocircuit [61,62]. Consequently, our results demonstrating that injury to most consciousness-related subcortical white matter microstructure was more severe in the DOC group than in the non-DOC group at the chronic stage could provide evidence for the loss of the complex interplay of diverse consciousness processes in the DOC group after DAI.

Several TBSS-based studies have described relationships between consciousness and white matter integrity at the chronic stage in TBI patients [63,64,65]. In 2009, Perlbarg et al. reported lower FA values of the cerebral peduncle, posterior limb of internal capsule, and posterior corpus callosum in the unfavorable outcome group (death, persistent vegetative state, minimally conscious state; Glasgow outcome score 1–3) compared to those in the favorable outcome group (minor or no residual impairments) at one year after onset in severe TBI patients [63]. In 2014, Sorg et al. demonstrated that ventral prefrontal white matter integrity was more degraded in mild TBI patients with loss of consciousness than in mild TBI patients without loss of consciousness using radial and axial diffusivity values at two to four years after the onset [64]. In 2018, Wang et al. reported correlations between the CRS and FA values of the pontine crossing tract, fornix, posterior limb of the internal capsule, and uncinate fasciculus at 2 months after onset in patients with disorders of consciousness (including hypoxic–ischemic encephalopathy, intracranial hemorrhage, and TBI) [65]. To the best of our knowledge, this is the first study to report on the relationship between impaired consciousness and whole subcortical white matter integrity at the chronic stage of DAI.

Nevertheless, this study had several limitations. First, information loss and the potential presence of artifacts could occur because TBSS analysis reduces white matter tracts to a skeleton framework, and along that projection, only the highest-valued FAs are projected to the center [43]. Second, the unbalanced sex distribution, notably the complete absence of females in the non-DOC group, introduces a potential bias. This zero-variance restricted the inclusion of sex as a statistical covariate, as it would distort parameter estimates. As a preliminary exploration to support this discussion, we assessed sex differences exclusively within the control group, which showed no substantial sex-based differences in global FA. However, a critical limitation of this assessment is that it ignores the possible interaction between sex and the disease state (DOC or non-DOC condition), which cannot be accounted for due to the lack of females in the patient groups. Consequently, this demographic limitation may affect the generalizability of our findings. Third, although our results showed severe damage to the extensive subcortical white matter microstructure in the DOC group, we could not perform a correlation analysis between the CRS-R score and the FA values of whole subcortical neural structures due to the small sample size. Hence, further prospective studies addressing these limitations in larger, strictly sex-matched cohorts are needed.

## 5. Conclusions

In conclusion, this study examined the relationship between impaired consciousness and white matter integrity in DAI patients by using TBSS-based analysis. In DAI patients, the state of consciousness was associated with injury severity across most subcortical neural structures. Consequently, the results suggest that the severity of impaired consciousness can indicate the severity of injury to the broad subcortical white matter structures in DAI patients. Hence, further studies are needed to analyze the state of the whole brain, including the various cortical-subcortical and cortico-cortical networks related to consciousness after DAI.

## Figures and Tables

**Table 1:** The demographic data of each group.

	DOC Group	Non-DOC Group	Control Group
Age (years)	52.55 ± 16.24	43.19 ± 14.96	41.73 ± 12.36
Number (*n*)	11	16	22
Male:Female	8:3	16:-	11:11
Duration to DTI (month)	5.68 ± 6.78	7.81 ± 6.12	-
GCS score (onset)	5.00 ± 1.34	6.06 ± 2.21	-
CRS-R score (onset)	2.36 ± 1.80	5.38 ± 4.05	-
CRS-R score (chronic)	15.64 ± 4.34	23	-

DOC: disorder of consciousness, DTI: diffusion tensor imaging, GCS: Glasgow coma scale, CRS-R: Coma Recovery Scale-Revised; Values are mean ± standard deviation.

**Table 2:** Comparison of the fractional anisotropy values between each group.

	DOC Group	Non-DOC Group	Control Group	*F*	*p*
Middle cerebellar peduncle ^abc^	0.38 ± 0.03	0.41 ± 0.02	0.46 ± 0.02	56.590	0.00 *
Pontine crossing tract ^abc^	0.36 ± 0.04	0.39 ± 0.03	0.42 ± 0.02	21.522	0.00 *
Genu of corpus callosum ^abc^	0.41 ± 0.05	0.46 ± 0.04	0.53 ± 0.02	50.353	0.00 *
Body of corpus callosum ^abc^	0.40 ± 0.06	0.47 ± 0.05	0.57 ± 0.03	59.101	0.00 *
Splenium of corpus callosum ^abc^	0.54 ± 0.06	0.60 ± 0.04	0.67 ± 0.02	47.356	0.00 *
Fornix (column and body) ^bc^	0.21 ± 0.04	0.26 ± 0.07	0.39 ± 0.08	29.630	0.00 *
Rt. corticospinal tract ^b^	0.40 ± 0.05	0.43 ± 0.04	0.46 ± 0.03	9.971	0.00 *
Lt. corticospinal tract ^ab^	0.40 ± 0.06	0.46 ± 0.04	0.48 ± 0.03	13.017	0.00 *
Rt. medial lemniscus ^abc^	0.46 ± 0.04	0.51 ± 0.03	0.55 ± 0.02	29.073	0.00 *
Lt. medial lemniscus ^abc^	0.46 ± 0.04	0.51 ± 0.03	0.56 ± 0.03	30.406	0.00 *
Rt. Inferior cerebellar peduncle ^abc^	0.36 ± 0.04	0.39 ± 0.02	0.41 ± 0.03	9.813	0.00 *
Lt. Inferior cerebellar peduncle ^abc^	0.36 ± 0.04	0.39 ± 0.02	0.41 ± 0.03	10.216	0.00 *
Rt. superior cerebellar peduncle ^bc^	0.41 ± 0.06	0.45 ± 0.04	0.52 ± 0.02	30.558	0.00 *
Lt. superior cerebellar peduncle ^abc^	0.40 ± 0.05	0.44 ± 0.03	0.50 ± 0.02	41.450	0.00 *
Rt. cerebral peduncle ^abc^	0.49 ± 0.05	0.54 ± 0.03	0.61 ± 0.03	45.045	0.00 *
Lt. cerebral peduncle ^abc^	0.49 ± 0.04	0.55 ± 0.03	0.62 ± 0.03	76.093	0.00 *
Rt. anterior limb of internal capsule ^abc^	0.41 ± 0.03	0.45 ± 0.02	0.50 ± 0.02	56.724	0.00 *
Lt. anterior limb of internal capsule ^abc^	0.40 ± 0.04	0.43 ± 0.02	0.50 ± 0.02	46.572	0.00 *
Rt. posterior limb of internal capsule ^abc^	0.50 ± 0.03	0.54 ± 0.02	0.59 ± 0.02	50.780	0.00 *
Lt. posterior limb of internal capsule ^abc^	0.51 ± 0.03	0.55 ± 0.02	0.61 ± 0.02	65.743	0.00 *
Rt. retrolenticular part of internal capsule ^abc^	0.45 ± 0.03	0.48 ± 0.02	0.51 ± 0.02	20.768	0.00 *
Lt. retrolenticular part of internal capsule ^bc^	0.46 ± 0.04	0.48 ± 0.02	0.52 ± 0.02	21.699	0.00 *
Rt. anterior corona radiata ^abc^	0.29 ± 0.04	0.34 ± 0.03	0.41 ± 0.04	41.083	0.00 *
Lt. anterior corona radiata ^abc^	0.29 ± 0.05	0.34 ± 0.03	0.41 ± 0.03	41.244	0.00 *
Rt. superior corona radiata ^abc^	0.35 ± 0.04	0.39 ± 0.02	0.44 ± 0.03	34.009	0.00 *
Lt. superior corona radiata ^abc^	0.36 ± 0.03	0.40 ± 0.02	0.44 ± 0.03	44.201	0.00 *
Rt. posterior corona radiata ^abc^	0.35 ± 0.03	0.39 ± 0.02	0.44 ± 0.02	51.979	0.00 *
Lt. posterior corona radiata ^abc^	0.34 ± 0.03	0.38 ± 0.03	0.43 ± 0.02	45.481	0.00 *
Rt. posterior thalamic radiation (includes optic radiation) ^abc^	0.42 ± 0.06	0.47 ± 0.04	0.53 ± 0.03	27.971	0.00 *
Lt. posterior thalamic radiation (includes optic radiation) ^abc^	0.42 ± 0.06	0.47 ± 0.04	0.53 ± 0.03	29.489	0.00 *
Rt. sagittal stratum ^abc^	0.38 ± 0.03	0.42 ± 0.03	0.48 ± 0.03	45.427	0.00 *
Lt. sagittal stratum ^abc^	0.35 ± 0.05	0.40 ± 0.03	0.45 ± 0.02	48.883	0.00 *
Rt. external capsule ^abc^	0.31 ± 0.02	0.33 ± 0.02	0.37 ± 0.02	40.641	0.00 *
Lt. external capsule ^abc^	0.32 ± 0.03	0.34 ± 0.01	0.38 ± 0.02	31.620	0.00 *
Rt. cingulum (cingulate gyrus) ^abc^	0.34 ± 0.03	0.38 ± 0.03	0.44 ± 0.03	43.468	0.00 *
Lt. cingulum (cingulate gyrus) ^abc^	0.36 ± 0.05	0.41 ± 0.03	0.47 ± 0.03	38.626	0.00 *
Rt. cingulum (hippocampus) ^bc^	0.28 ± 0.04	0.30 ± 0.03	0.36 ± 0.03	35.404	0.00 *
Lt. cingulum (hippocampus) ^abc^	0.28 ± 0.04	0.32 ± 0.02	0.37 ± 0.03	31.843	0.00 *
Rt. fornix (crus) ^abc^	0.30 ± 0.06	0.36 ± 0.03	0.43 ± 0.04	39.793	0.00 *
Lt. fornix (crus) ^abc^	0.34 ± 0.06	0.40 ± 0.04	0.47 ± 0.03	32.773	0.00 *
Rt. superior longitudinal fasciculus ^abc^	0.36 ± 0.03	0.39 ± 0.02	0.42 ± 0.02	36.045	0.00 *
Lt. superior longitudinal fasciculus ^abc^	0.37 ± 0.02	0.40 ± 0.02	0.43 ± 0.02	34.064	0.00 *
Rt. superior fronto-occipital fasciculus ^abc^	0.29 ± 0.05	0.35 ± 0.03	0.44 ± 0.04	50.839	0.00 *
Lt. superior fronto-occipital fasciculus ^abc^	0.27 ± 0.07	0.34 ± 0.03	0.42 ± 0.05	33.906	0.00 *
Rt. uncinate fasciculus ^abc^	0.34 ± 0.04	0.38 ± 0.03	0.44 ± 0.03	42.459	0.00 *
Lt. uncinate fasciculus ^bc^	0.33 ± 0.03	0.36 ± 0.03	0.43 ± 0.02	54.461	0.00 *
Rt. tapetum ^abc^	0.27 ± 0.04	0.31 ± 0.03	0.37 ± 0.03	29.544	0.00 *
Lt. tapetum ^abc^	0.23 ± 0.02	0.25 ± 0.02	0.28 ± 0.02	25.478	0.00 *

DOC: disorder of consciousness; Values are mean ± standard deviation. ^a^ DOC group significantly differs from Non-DOC group per Tukey HSD test, *p* < 0.05. ^b^ DOC group significantly differs from control group per Tukey HSD test, *p* < 0.05. ^c^ Non-DOC group significantly differs from control group per Tukey HSD test, *p* < 0.05. * *p* < 0.05.

## Data Availability

The datasets generated and analyzed during the current study are not publicly available due to institutional ethical policies and local data protection regulations regarding sensitive human neuroimaging and clinical data. However, a deidentified minimal dataset is available from the corresponding author upon reasonable request for scientific research purposes.
